# Fear and Coping with Death in Intensive Care Nurses: a Structural Model Predictor of Compassion Fatigue*

**DOI:** 10.17533/udea.iee.v41n1e12

**Published:** 2023-03-14

**Authors:** Josué Medina-Fernández, Nissa Yaing Torres-Soto, Karina Casco-Gallardo, Anahí Ruiz-Lara, Beatriz Martínez-Ramírez, Esmeralda Fuentes-Fernández

**Affiliations:** 1 Masters in Nursing. Professor and full-time Researcher. Universidad Autónoma del Estado de Quintana Roo, México.Email: josuemedinafernandez@outlook.es Universidad de Quintana Roo Universidad Autónoma del Estado de Quintana Roo Mexico josuemedinafernandez@outlook.es; 2 PhD in Social Sciences. Professor and full-time Researcher. Universidad Autónoma del Estado de Quintana Roo, México. E-mail: nissa.torres@uqroo.edu.mx. Universidad de Quintana Roo Universidad Autónoma del Estado de Quintana Roo Mexico nissa.torres@uqroo.edu.mx; 3 Masters in Nursing. Professor and full-time Researcher. Universidad Autónoma del Estado de Quintana Roo, México. Email: karina.casco@uqroo.edu.mx Universidad de Quintana Roo Universidad Autónoma del Estado de Quintana Roo Mexico karina.casco@uqroo.edu.mx; 4 Masters in Nursing. Professor and full-time Researcher. Universidad Autónoma de Coahuila, México. Email: anahi.ruiz@uadec.edu.mx. Universidad Autónoma de Coahuila Universidad Autónoma de Coahuila Mexico anahi.ruiz@uadec.edu.mx; 5 Masters in Health Sciences. Professor and full-time Researcher. Universidad Autónoma del Estado de Quintana Roo, México. Email: beatriz.martinez@uqroo.edu.mx Universidad de Quintana Roo Universidad Autónoma del Estado de Quintana Roo Mexico beatriz.martinez@uqroo.edu.mx; 6 PhD in Education Sciences. Professor and full-time Researcher. Universidad Autónoma del Estado de Quintana Roo, México. Email: esmefuentes@uqroo.edu.mx Universidad de Quintana Roo Universidad Autónoma del Estado de Quintana Roo Mexico esmefuentes@uqroo.edu.mx

**Keywords:** fear, death, compassion fatigue, empathy, mental fatigue, critical care, nursing staff, miedo, muerte, fatiga por compasión, empatía, fatiga mental, cuidados críticos, personal de Enfermería, medo, morte, fadiga compaixão, empatía, fadiga mental, cuidados críticos, recursos humanos de enfermagem

## Abstract

**Objective.:**

To determine the effect of fear and coping with death on compassion fatigue in nurses working in the intensive care unit.

**Methods.:**

Correlational-predictive design, applied in 245 nurses working in the intensive care unit through intentional sampling. The study applied a personal data card, the Collet-Lester Fear of Death Scale (α=0.72), the Bugen Fell of Death Scale (α=0.82), and the Empathy Exhaustion Scale (α=0.80). Descriptive and inferential statistics were performed, such as Spearman's test and a structural equation model.

**Results.:**

The work had 255 nurses who participated, finding a relationship among fear and coping toward death and compassion fatigue (*p*<0.01), together with the equation model showing that fear and coping toward death have a positive effect in 43.6% on compassion fatigue.

**Conclusion.:**

Fear and coping with death have an effect on compassion fatigue in nurses working in the intensive care unit, so that when working in a critical area it can cause health effects.

## Resumen

**Objetivo.:**

Determinar el efecto del miedo y afrontamiento ante la muerte sobre la fatiga por compasión en enfermeros que laboran en la unidad cuidados intensivos.

**Método.:**

Diseño correlacional-predictivo. A través de un muestreo intencional se seleccionaron 255 enfermeros que laboraban en Unidades de Cuidados Intensivos de Adultos de hospitales de la Península de Yucatán (México). Se aplicó una cédula de datos personales, la escala de miedo a la muerte de Collet-Lester (α=0.72), la escala de Bugen de afrontamiento de la muerte (α=0.82) y la escala de agotamiento por empatía (α=0.80). Se realizó estadística descriptiva e inferencial como prueba de Spearman y un modelo de ecuación estructural.

**Resultados.:**

Se encontró relación del miedo y el afrontamiento hacia la muerte con la fatiga por compasión (*p*<0.01). Adicionalmente, el modelo de ecuaciones muestra que el miedo y el afrontamiento hacia la muerte predice en un 43.6% sobre la fatiga por compasión.

**Conclusión.:**

El miedo y el afrontamiento hacia la muerte tienen efecto sobre la fatiga por compasión en los enfermeros que laboran en la unidad de cuidados intensivos, por lo que al estar laborando en esta área crítica puede provocar afectaciones en su estado de salud.

## Resumo

**Objetivo.:**

Determinar o efeito do medo e enfrentamento da morte na fadiga por compaixão em enfermeiros que atuam em Unidade de Terapia Intensiva (UTI).

**Métodos.:**

Desenho preditivo correlacional. Por meio de amostragem intencional, foram selecionados 255 enfermeiros que trabalhavam em Unidades de Terapia Intensiva de Adultos de hospitais da Península de Yucatán (México). Foi aplicada uma ficha de dados pessoais, a escala de medo da morte de *Collet -Lester* (α=0.72), a escala de Bugen de enfrentamento da morte (α=0.82) e a escala de exaustão por empatia (α=0.80). Foram realizadas estatísticas descritivas e inferenciais como o teste de *Spearman* e um modelo de equação estrutural.

**Resultados.:**

Encontrou-se relação entre medo e enfrentamento da morte e fadiga por compaixão (*p*<0.01). Além disso, o modelo de equação mostra que o medo e o enfrentamento da morte preveem 43.6% da fadiga por compaixão.

**Conclusão.:**

O medo e o enfrentamento da morte afetam a fadiga por compaixão em enfermeiros que atuam em UTI, portanto, trabalhar nessa área crítica pode afetar seu estado de saúde.

## Introduction

In the nursing science, the fundamental role of the profession is based on the prevention, care, treatment, rehabilitation, and management of processes, and in this entire area of competence, the sadness of people is also experienced. Hence, it is important for the nursing staff to have strategies that allow them to intervene in a holistic, qualitative and personal manner, as well as take care of themselves mentally, with special attention in cases of mortality during the working day. Death is a natural, universal and indivisible fact of the human condition; it is an inevitable consequence of life, includes biological, social, cultural, and psychological aspects, and perhaps this is one of the events that has the greatest impact on the emotions of the individuals themselves and on their personal social network, their needs and those of individuals who care about the deceased.^1^

In intensive care, part of the nursing practice is to try to prepare to live death (their own and that of others) with human sensitivity, which can be taboo for professionals working in health institutions; this can be a reason for reflection and, in turn, cause fear, anguish, and little coping about the end of life.^2^ Thus, nursing professionals cope with the suffering of hospice patients and their families throughout their working lives, which can generate fear on the face of imminent death,^3^ likewise, it should be highlighted that nurses have more contact with patients than any other health professional; therefore, both the fear of death and the ability to cope can affect the quality of care and increase mental fatigue.

It has been argued that the nursing staff closest to the grief and death process experience changes in their physical and emotional status due to the influence of stress, which leads to changes in their overall health that affect the quality of life, as well as in the sensitivity, capacity, and attitude toward patients who are suffering (either loss of health or chronic and ongoing illness) and the probable presence of compassion fatigue (CF). For terms, herein, CF is conceptualized as a response to prolonged exposure to emotional and interpersonal stressors at work^4^ manifested by changes in the psychosocial state due to empathic exposure to trauma experienced by others, manifested by diminished enthusiasm, sadness, irritability, and extreme fatigue. It is also expressed by absenteeism and low productivity, leading to the desire to quit a job or even a career. The term was coined specifically for health professionals as a "subclassification" of the burnout syndrome. In this syndrome, wear is greater with the lack of ability to deal with physical and environmental threats. 

With respect to the frequency of CF in the nursing staff, it is indicated that in the hospital area, at least 25% of professionals experience high risk of developing compassion fatigue and 50% moderate risk. In other words, nearly 80% of all the nursing staff has a moderate-high risk of developing CF.^5^

Graystone^6^ indicates that CF and burnout are two of the most-serious problems faced by the nursing profession currently, reinforces that the consequences of these problems are related with mental fatigue, professional frustration, job disappointment, low productivity and poor quality of care. It should be remembered that, given the culture and values of the profession, many signs and symptoms are masked, often affecting those who serve the longest. Factors influencing on the start and/or severity of the problem include excessive nursing care, especially for long-term critically ill patients, when staff are perceived as “incompetent” or “frustrated” at not having the ability to cure or soothe their suffering.^7^ This continuous care of patients in the critical area, as well as the fear of death, poor coping with it and suffering due to complicated illness of terminally ill and moribund patients and their families, together with responsibilities in daily life, their own burdens and worries, along with low training in managing emotions and effective coping strategies can wear down their defenses, affect their integrity and mental health.^7^^,^^8^

Nevertheless, CF is a phenomenon present in the health setting, especially in those working in hospital units with more severe patients or patients in prolonged treatment, such as intensive therapy, internal medicine, emergency, among others.^4^^-^^6^^,^^9^^,^^10^ Independent of their condition, the nursing staff caring for people with serious health conditions must emphasize on the impact and must be educated regarding its awareness, of the risk factors and - quite important - the alternatives for prevention and/or management of the problem discussed. It is necessary to be aware of the existence of the problem, of the factors associated, and timely recognition of the initial symptoms, given that it constitutes one of the keys for early diagnosis and treatment of CF. Considering the problem described, the aim of this study was to determine the effect of fear and coping with death on compassion fatigue in nurses working in the adult intensive care unit.

## Methods

*Design.* Design with correlational-explanatory type quantitative approach, seeking to determine if the independent variables are affected over the dependent, that is, if one variable influences over the other.^11^

*Setting.* Data was collected from nurses working in an adult intensive care unit from a public or private hospital in the Yucatan peninsula comprised by the states of Campeche, Quintana Roo, and Yucatan (Mexico).

*Subjects.* Using the G Power sample calculator program, a sample size of 160 nurses was obtained, considering a finite sample with 0.05 probability of making type 1 error, power of 90% (1-β=0.9), effect size of 0.10. The sample collected was of 255 nurses, exceeding the initial size expected. For the study’s viability, an intentional sampling was carried out in which nurses whose seniority was ≥ 1 year working in an adult intensive care unit of a public and private hospital in the Yucatan peninsula were considered to participate, with at least a nursing technician degree and having experienced the death of at least one patient while in their care. Among the exclusion criteria were those without permanent work assignment in the ICU and who do not have direct care of someone in the ICU.

*Data collection.* Data were collected by research aides, who had been trained via virtual means to apply the instruments through social media (WhatsApp, Facebook, Instagram, and Twitter). Among the instruments used, first, a personal data card was applied, which inquired about data, like age, sex, marital status, last academic degree, years working, and type of hospital where they worked. Thereafter, the Collet-Lester fear of death scale (CL-FODS) was applied, which evaluates attitudes about death, especially differentiating between death itself and the dying process, referring to one's own death and death. This version, adapted into Spanish, is made up of four subscales that provide multidimensional information about the fear of one’s own death, fear of one’s own dying process, fear of the death of others, and fear of the dying process of others. It contains 28 items, grouped into four subscales with seven items each. The responses are Likert type, where 1 means “not at all” and 5 means “very much”, mentioning that the total scores of the scale vary between a minimum of 28 and a maximum of 140. The highest mean scores indicate greater fear of death or of the dying process, demonstrating internal consistency of α = 0.72.^12^

To measure coping with death, Bugen’s Coping with Death Scale was applied, which measures nurses' competency in managing death and their knowledge related with its preparations, and it is made up of 30 items, with Likert-type answers, with options from 1 (“totally disagree”) to 7 (“totally agree”). Results can range from 30 to 210 points, where it is interpreted that the higher the score, the better the skills for coping with death. When scoring below the 33^rd^ percentile, it indicates low coping; above the 66^th^ percentile, it corresponds to high coping. When scoring in the intermediate zone, neutral coping is evidenced. The items obtained a Cronbach’s alpha coefficient of 0.824.^13^ Lastly, to evaluate CF, the Compassion Fatigue Scale (CFS) was applied, which permits obtaining diagnoses on empathetic behavior and the risk of contracting compassion fatigue. It is a multidimensional questionnaire comprised of three dimensions: 1) Vulnerability: this dimension indicates the extent to which professionals feel affected by their work at physical, psychological, emotional, or social levels; 2) Personal care resources: this dimension indicates the extent to which professionals have personal and technical resources to deal with the bio-psycho-physical exhaustion resulting from relational work; and 3) Personal involvement: this dimension evaluates the degree of involvement in the relationship between professionals and their clients. The instrument is made up of 27 items, evaluated as a Likert scale with four possible response options (0- fully agree, 1- agree. 2- disagree, and 3- fully disagree), with a Cronbach’s alpha coefficient of 0.80 and is interpreted that the higher the score, the greater the CF.^14^

*Data analysis.* Data were analyzed with the SPSS statistical package version 26. Descriptive statistics were used, obtaining absolute frequencies and percentages for categorical variables, measures of central tendency and variability for numerical variables. A distribution analysis of the continuous variables was performed with the Kolmogorov Smirnov test to determine the normality of the variables, applying Spearman’s test for the correlation among the variables. A structural equation model was tested using the statistical software EQS v6.1. The chi-square (χ2) will be the statistical indicator; if this relationship results with a significance level of p < 0.05, it will be considered that the model presents an adequate statistical fit. Considering that χ^2^ tends to be susceptible to the sample number, the relative χ2 will be used, which is calculated by dividing the adjusted χ2 index by the degrees of freedom. If this value is < 5, it Will be considered a good statistical fit. Additionally, given that the statistical indicators tend to be quite sensitive to the sample size, the following were used: the Comparative Fit Index (CFI), Bentler-Bonett Norm Fit Index (BBNFI) and the Bentler-Bonett Non-Norm Fit Index (BBNNFI) ≥ 0.90, and the Root Mean Square Error Approximation (RMSEA), which is an absolute population fit mean with value ≤ 0.09.

*Ethical considerations.* The research proposal was approved by the ethics committee in the “Dr. Santiago Valdés Galindo” Faculty of Nursing at Universidad Autónoma de Coahuila, complying with that established in the regulation of the General Health Legislation on research, Article 13 of Chapter 1, second title was applied, treating participants with respect and protecting their well-being; clearly explaining the objective of the study and all activities or procedures carried out in the investigation. This was fulfilled through the delivery and signing of the informed consent, where the human rights of the participants were protected, along with their autonomy, with the right to free decision, which involves collecting and evaluating their data, respecting confidentiality and anonymity if desired, without the intention of causing any discomfort or harm to the study subjects at any given moment. Hence, reference was made to the General Health Legislation in its second title "On the ethical aspects of research on human beings" with articles 13, 17, 18, 20, 21. This legislation addresses the subject of study as a being, where the criteria of respect, dignity and protection of rights and well-being must prevail. The study was considered of minimum risk and was cancelled when there was damage to health, in addition to the fact that informed consent must be applied, explained clearly and precisely. Likewise, the Declaration of Helsinki and respect for the principles of justice, beneficence, respect and non-maleficence of the Belmont report were considered.

## Results

The study had participation by 255 nurses who worked in the ICU, with a mean age of 34.41 years, *SD* = 8.882 years, having a labor seniority mean of 9.48 years, *SD* = 6.804 years, finding that most were women, with single marital status, with a maximum study degree of bachelor's degree in nursing and working in a public hospital (Table 1).

**Table 1:** Description of the 255 study participants

Variable	Frequency	%
*Sex*		
Male	82	33.5
Female	163	66.5
*Marital status*		
Single	99	40.4
Married	95	38.8
Divorced	10	4.1
Common law	34	13.9
Other	7	2.9
*Level of studies*		
Technician in Nursing	63	25.7
Bachelor’s in Nursing	111	45.3
Specialist Nurse	57	23.3
Master's Nurse	14	5.7
*Type of hospital*		
Public	211	86.1
Private	34	13.9

Based on the overall scores of the principal variables studied, 2% (5) has low fear of death; 45.3% (111) neutral fear; and 52.7% (129) high fear of dying. Regarding coping, it was found that 46.1% (113) had low coping; 49% (120) neutral coping; and 4.9% (12) high coping with death. Table 2 describes the scores obtained from the three instruments, finding that the three variables are below the established mean. 

**Table 2:** Description of the scores for the scales of fear of death, coping with death, and compassion fatigue

Variable	*M*	*SD*	*Max. value*	*Min. value*	*95% CI*
*Fear of death*	96.40	1.497	140	28	99.45 - 93.45
Own death	21.81	0.483	35	7	20.86 - 22.76
Own dying process	23.89	0.476	35	7	22.96 - 24.83
Death of another	26.34	0.429	35	7	25.49 - 27.18
Other’s dying process	24.35	0.428	35	7	23.51 - 25.19
*Coping with death*	108.37	1.889	190	42	104.65 - 112.09
*Compassion fatigue*	34.87	0.444	52	9	34.00 - 35.75
Vulnerability	11.66	0.214	20	1	11.24 - 12.08
Personal care	11.48	0.213	20	3	11.06 - 11.90
Professional involvement	11.73	0.150	19	6	11.44 - 12.03

Note: M = Mean, SD = Standard deviation, Max value = Maximum value, Min value = Minimum value, 95% CI = 95% confidence interval

To determine the relationship of the study variables, a Spearman correlation was performed, finding relationship between fear of death and coping with death with compassion fatigue (*p* < 0.01), that is, the greater fear and coping with death the greater compassion fatigue felt by the ICU nurses. Similarly, relationship was found of fear of death with coping with death (*p* < 0.01), meaning that with greater fear there is greater coping with death (Table 3). 

**Table 3:** Correlation values among the variables studied

Variable	1	2	3	4	5
1. Age	1	-0.038	-0.078	0.040	-0.104
2. Years worked		1	-0.059	0.022	-0.075
3. Fear of death			1	0.380**	0.303**
4. Coping with death				1	0.234**
5. Compassion fatigue					1

Note: * *p* < 0.05, ** *p* < 0.001

**Figure 1:**
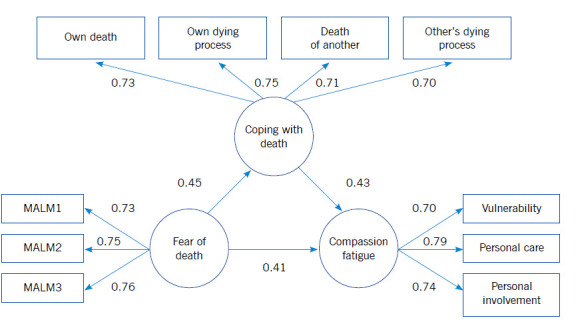
Structural model of variables affecting compassion fatigue. Note: All structural coefficients and factorial weights were significant (*p* < 0.05). Goodness of fit: N = 245, χ^2^ = 203.88 (*g.l.*= 39), *p* = 0.28, *BBNFI* = 0.97, *BBNNFI* = 0.98, *CFI* = 0.99, *RMSEA* = 0.03. R^2^ = 0.43.

Figure 1 shows the results of the structural model tested. High and significant factor loadings are revealed (*p* < 0.05) in the variables manifested corresponding to the latent variables of coping with death, fear of death and compassion fatigue. The results showed that fear of death affects positively and significantly compassion fatigue (0.41) and coping with death (0.45); in turn, coping with death affects positively and significantly compassion fatigue (0.43). The structural model achieved its acceptance because it complies with suitable statistical (χ2 = 203.88 [g.l.= 39], p = 0.28), practical (BBNFI = 0.97, BBNNFI = 0.98, CFI = 0.99), and population (RMSEA = 0.03) indicators. The R^2^ index of the compassion fatigue variable indicates the model explains 43% of the variance of the construct, which may be considered moderate, according with the criteria described by Chin (1998) for values of R^2^. Thereby, it is concluded that the model has some explanatory power for the factor “compassion fatigue”.

## Discussion

As first approach in this work, it is necessary to analyze the characteristics of the study population because it appears to follow the same pattern at the national and international level, given that the majority are women and have a maximum bachelor's degree education level. This is due to two important aspects in Mexico, the first is that from the cultural perspective, women have been the main innate caregivers during childhood, adolescence, adulthood, and old age, so this line of thinking is often applied in the nursing sciences inasmuch as it was considered an eminently feminine activity and emphasized in its beginnings as a profession since 1860 when Florence Nightingale established the bases of said science. The second is due to the fact that professionalization in nursing has been a precursor for improving the quality of care during the last decade, which is why there has been an increase in level of education, leaving as a challenge the increase of nurses with graduate degrees.^15^^,^^16^

With respect to the variable of fear of death, a mean of 96.40 points was found, which is lower than that found in Spain, Costa Rica, and Peru. This is because emotional regulation is provided by clinical experience and training in the care of critically ill patients, which could be influencing fear of death. Given the foregoing, our study provides a new contribution to nursing knowledge since most registered documents focus on nursing students or the health area, coupled with the fact that direct evidence on intensive care nursing staff is minimal or null, making it necessary to delve into this theme in active professionals in clinical areas.^17^^-^^19^ In turn, coping with death in this study had a mean of 108 points, which is lower than that found in countries, like Colombia, Costa Rica, and Spain. This demonstrates that coping was better in these countries because the nursing staff uses coping strategies focused on emotions, inhibiting feelings towards patients the families, using communication and prayer with patients, as well as accompaniment to soothe the families’ suffering. ^(^^17^^-^^20^

Furthermore, in the variable of compassion fatigue it was found that it has been addressed from an oncological perspective, in emergency, palliative care, but to a lesser extent in intensive care, highlighting that the main countries that have investigated it are Brazil and Colombia,^21^^-^^23^ which is why this study took on the opportunity to evaluate it with another instrument, finding high scores above the mean due to the fact that - as nurses - care with empathy and the capacity for interpersonal relationships are vital parts of the profession, so compassion contains an internal conviction and resistance that transmits the message "I understand you and I feel you", and although compassion is valued in the nursing profession, it can also lead to emotional and work-related problems.^24^ Similarly, this research found that the greater the fear and coping with death, the greater compassion fatigue the ICU nurses have. These results are justified because they are factors that develop according to experience, and if nursing professionals have not had them, this mental exhaustion considered as compassion fatigue increases,^25^ highlighting in this research greater fear of dying and low coping with death, being an area of interest to analyze these correlations with other articles; however, it has not been analyzed or considered in specific areas, like the intensive care unit.

Lastly, fear and coping with death predict compassion fatigue by 43.6%; this is justified because the nursing staff do not recognize their vulnerability as persons. Therefore, as health professionals, they seek to avoid illness, complications, and death, presenting a reality that is difficult to address, in addition to the fact that nursing professionals approach death differently, given that they consider care as holistic. from a biological, psychological, social, and spiritual perspective, considering their culture and way of life.^22^^,^^26^

It is concluded that fear and coping with the user’s death predicts the risk of compassion fatigue in nurses working in the intensive care units, therefore, working in a critical area can affect mental health, making it necessary to perform nursing interventions that help to diminish this fatigue by applying resilience, self-care, and continuous hospital education that helps to control the affected variable in the physical, psychological, social, and spiritual domains. 

The information introduced in this research can also contribute to the formulation of public policies in several countries throughout the world. It can be applied to personnel working in health care institutions and in the educational field to train future nursing professionals who can develop these kinds of competencies, which in the future will be useful and will provide health throughout their professional career. Death, pain, loss, to mention some factors, are elements with which health professionals live daily to a greater or lesser extent according to where they work. In this sense, the nursing staff conduct their activities in diverse settings, which will possibly confer wear and compassion fatigue, hence, knowing the predictors that will allow developing personal and work strategies to prevent it, can contribute to better performance or formulating institutional strategies for human resources to develop their skills and competencies optimally. 

Among the study limitations, difficulty was found in collecting data in the diverse public and private institutions, coupled with the lack of precise data on the number of nurses in the intensive care units in the Yucatan peninsula. It is suggested to keep a finite number for sample identification.
